# Enhancing food access in a comprehensive cancer center area of influence through local partner capacity building

**DOI:** 10.1002/cam4.70070

**Published:** 2024-08-17

**Authors:** Preena Loomba, Margaret R. Raber, Mayra Aquino, Nikki Rincon, Lori Rumfield, Karen M. Basen‐Engquist, Ruth Rechis

**Affiliations:** ^1^ Department of Health Disparities Research University of Texas MD Anderson Cancer Center Houston Texas USA; ^2^ Cancer Prevention and Control Platform University of Texas MD Anderson Cancer Center Houston Texas USA; ^3^ Hearts and Hands of Baytown Baytown Texas USA; ^4^ Goose Creek Consolidated Independent School District Baytown Texas USA

**Keywords:** cancer disparity, community outreach and engagement (COE), food insecurity

## Abstract

**Background:**

Food insecurity, an economic and social condition of limited food access, is associated with poor diet quality—a risk factor for several common cancers. The University of Texas MD Anderson Cancer Center supports healthy food access through community‐led evidence translation by actively partnering with community‐based organizations (CBOs). These partnerships aim to enhance the capacity of food assistance CBOs to effectively implement evidence‐based food insecurity mitigation programs in the cancer center's area of influence.

**Methods:**

This case study aims to describe the cancer center's model for local food access capacity building and detail operationalization in the context of a whole‐community cancer prevention effort (Be Well Baytown) in Baytown, Texas.

**Results:**

Elements central to the capacity building model include (i) assessment of baseline needs and capacity, (ii) empowering a community champion within a relevant CBO, (iii) mapping inter‐sectoral community partnerships, collaborations, and linkages, and (iv) leveraging systems, connections, and resources to provide an enabling environment for overall food access systems growth. Through this process, Be Well Baytown enhanced the capacity of a local food pantry leading to increases in total reach, pounds of food distributed, and number of food distribution events in collaboration with intersectoral partners from 2018 to 2023.

**Conclusion:**

This case study highlights the model's implementation as a co‐benefit community partnership strategy to maximize the impact of food security programs integrated with comprehensive cancer center prevention efforts.

## Abstract 2

The purpose of this case study is to summarize learning from supporting food assistance CBOs to effectively implement evidence‐based food insecurity mitigation programs and to better understand how to optimize use of community‐driven food security programs aligned to Comprehensive Cancer Center's (CCC) community outreach and engagement (COE) efforts. The case study demonstrates a successful community partnership strategy integrating food security programs into comprehensive cancer prevention efforts that improved the capacity of a local food pantry, resulted in expanded reach, increased food distribution, and more collaborations with partners from 2018 to 2023 offering a model for other CCCs.

## 1 INTRODUCTION

Long‐standing social, economic, environmental, and structural disparities contribute to differential impact that poor health has across populations, and health outcomes improve with increasing socioeconomic status (SES).^1^, ^2^ Social determinants of health (SDOH) refer to the social factors and the unequal distribution of these factors that contribute to health inequities. SDOH such as food, housing, and transportation insecurities have been linked to cancer risk and outcomes disparities.^3^ More than one in five individuals with cancer struggle to meet at least one of these needs, and the numbers are even higher for patients with cancer who are Black, Latino, or live in low income households.^4^, ^5^, ^6^, ^7^

Food insecurity (FI), or lack of access to nutritionally adequate foods, is highly relevant to cancer prevention, treatment, and survivorship. Nearly 12.8% of all households in the United States experienced FI at some point in 2021.^8^ The experience of FI is more severe in low‐income communities, and for those already experiencing poor health.^9^ FI is associated with poor diet quality and increased risk for diet‐attributable diseases including obesity, hypertension, cardiovascular disease, and type 2 diabetes.^10^ Sub‐optimal diet was estimated to contribute to over 3 million new cancer cases among the US adults over a lifetime, or 7.4% of all new cancers^11^ For cancer survivors, FI during and after treatment can create mental and physical health challenges to navigating care and survivorship.^12^ Additionally, high out‐of‐pocket expenses often force choices between paying for treatment or affording nutritious food, increasing the risk of FI, which further highlights the need for cancer centers to address FI in their areas of influence.

Promoting healthy eating may reduce cancer risk and improve survivor wellness,^13^, ^14^ those experiencing FI may face practical barriers to implementing dietary recommendations.^15^, ^16^ Community‐engaged approaches to addressing SDOH, including FI, are being increasingly prioritized by NCI‐designated Comprehensive Cancer Centers (CCC).^12^ The National Cancer Institute (NCI) requires that community outreach and engagement (COE) efforts be integrated throughout the operations of CCCs, emphasizing the importance of working with community stakeholders to identify and address upstream health needs of the center's area of influence. CCCs have the potential to lead food security efforts through their COE programming, but integrating food access programs into CCC organizational structure comes with practical challenges.

Food provision to address scarcity is outside the current scope of many tertiary cancer centers, highlighting the importance of partnerships with community‐based organizations (CBOs) that can bring critical infrastructure, population access, and program expertise.^12^ Engaging community partners for public health efforts promotes communication and trust between communities and health systems, generates innovation, and reduces health inequities.^17^, ^18^ There is an opportunity for CCCs to build up existing community assets for food security through investment of COE resources. Meaningful and effective capacity building investments can benefit food assistance CBOs, such as local food pantries, to become more cohesive, adaptive, and better placed to confront economic, environmental, and social challenges related to food security.

The University of Texas MD Anderson Cancer Center (MD Anderson) located in Houston, Texas, one of 53 CCCs nationwide, is undertaking programs that combine patient care, research, and prevention. Be Well Communities™ is a core program of the Cancer Prevention and Control Platform at MD Anderson and is the institution's place‐based strategy for comprehensive cancer prevention and control, working with communities to address modifiable risk factors for cancer.^19^, ^20^, ^21^ Be Well Communities conducts community assessments and engages a coalition of community organizations from multiple sectors (the Steering Committee) to plan, implement, evaluate, and sustain evidence‐based interventions for cancer prevention in defined communities. Three Be Well Communities have been initiated thus far.^20^, ^21^ Detailed elsewhere,^19^ Be Well™ Baytown was launched in 2016 in Baytown, Texas, the third largest city in Harris County. Baytown has a population of 86,000 residents with 53.5% identifying as Hispanic or Latino, 12.2% as African American,^22^ 20.7% are foreign‐born,^23^ 24.9% are not covered by health insurance,^24^ and 19.5% are living below the federal poverty line.^25^ One in five (21.7%) Baytown households received food stamps or benefitted from the Supplemental Nutrition Assistance Program (SNAP) in 2022.^26^ Based on the Food Insecurity Index,^27^ a measure of food accessibility correlated with economic and household hardship that organizes areas in the United States by giving an index value from 0 (low need) to 100 (high need), most zip codes in Baytown have values over 80 in 2019, suggesting high need in the community for food insecurity mitigation.

The objective of this case study is to describe the Be Well Communities approach to enhancing local capacity to improve food access. Then, we detail the model's operationalization through Be Well Baytown, tracking the capacity and reach of one local food pantry over the first 6 years of program, which included years before and during the COVID‐19 pandemic. Finally, we discuss broader implications of the model as a co‐benefit community partnership strategy to drive food security initiatives aligned with CCC COE efforts.

## 2 METHODS

### 2.1 Be Well Communities model to build local capacity to enhance food access

**FIGURE 1:**
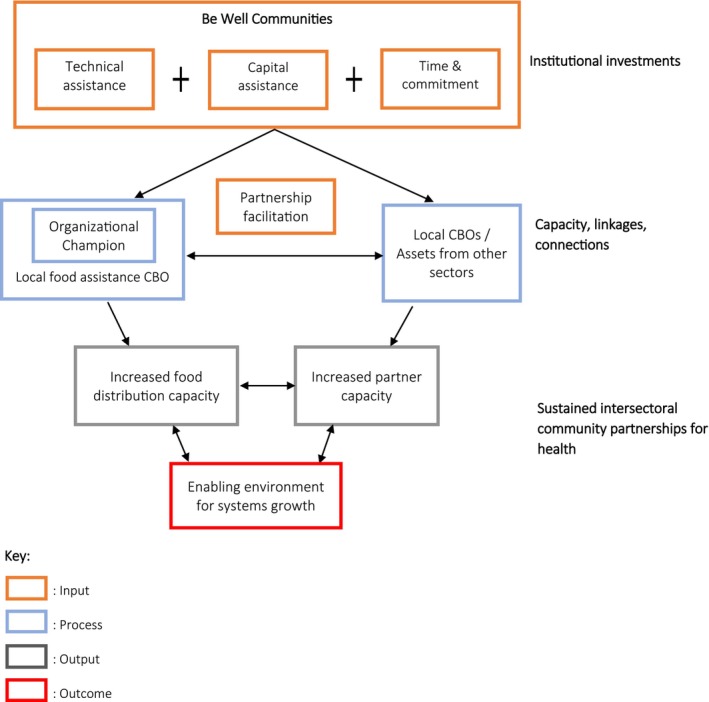
Be Well Communities Model for Building Community Capacity for Food Access.

Be Well Communities is centered around multi‐sector Steering Committees that implement programming in key health areas for cancer prevention.^19^ In the context of FI, local organizations that work on mitigation, such as food pantries, serve as key implementation partners for healthy eating programs and activities. The Be Well Communities model for local and systemic capacity building to enhance healthy food access is shown in Figure 1. The model's capacity building elements are consistent with the community capacity dimensions identified by Goodman et al. including: leadership, skills, resources, social and inter organizational networks, citizen participation, sense of community, community power, community values, and critical reflection.^28^ Thematically, the elements encompass the major inputs, processes, and outcomes to demonstrate efforts that enhance community capacity to expand food access. The capacity building elements of the model include: (1) assessing needs and capacity of local food access organizations, (2) empowering an individual as a champion within the community organization, (3) mapping inter‐sectoral community partnerships, collaborations, and linkages, and (4) leveraging systems, connections, and resources to provide an enabling environment for overall systems growth.

The primary input is the investment made by Be Well Communities to expand and strengthen structures that contribute to the capacity of local food access organizations and community organizations from other sectors that can function as partners (gray boxes in Figure 1). Technical assistance, partner facilitation, time commitment, and capital assistance are among the resources invested to establish and sustain program development. The linkages and inter‐dependency between the food access organizations and the partners from other sectors, defined as partnership facilitation, is another input in the model that contributes to creation of connections and cross‐sectoral collaborations. Partner facilitation is conceptualized as building an ecosystem of collaborative, multi sectoral partnerships. The arrows represent an iterative process to build community capacity for food security and illustrate the dynamic link between local food assistance CBOs, other partners, and Be Well Communities.

### 2.2 Model operationalization: Hearts and Hands of Baytown

The Be Well Communities model described above was operationalized through Be Well Baytown, with data collection on metrics of food assistance, CBO capacity, reach, and partnerships occurring between fiscal years 2018 and 2023.

#### 2.2.1 Local food assistance CBO

Hearts and Hands of Baytown (HHB), a ministry of Iglesia Cristo Viene, is a faith‐based organization with a mission of “alleviating the physical and emotional hunger of those in need in a dignified and supportive way”. HHB offers a range of food assistance programming (Table 1) and is the primary implementation partner for food access work undertaken as a part of Be Well Baytown.

**TABLE 1:** Programs implemented by Hearts and Hands of Baytown 2018–2023.

Hearts and Hands Programs	Data collection years	Description
Client Choice Fresh Market and Client Choice Nudges Program at 4 Pantries	2018–23	Fresh market offers supermarket‐style pantry where clients can freely choose items just as they would in a supermarket Nudges program distributes food, household, and personal items, with a focus on client confidence and dignity
Food for Change Fresh Market	2022–23	Food prescription program provides 60 lbs of fresh produce twice a month to individuals with chronic conditions Food scholarship program provides 60 lbs of fresh produce twice a month to individuals in educational programs
Doorstep Blessing	2022–23	Food‐delivery program that pairs volunteers with populations facing barriers to attending a traditional program (e.g., homebound individuals), implemented during the Covid‐19 pandemic The program also serves income‐eligible seniors (60+)
Mobile Food Fair events hosted by Hearts and Hands of Baytown	2018–23	Drive‐thru or walk‐up events offering fresh produce, meat, milk, and assorted groceries Includes Paula's Pantry, an outreach program for individuals experiencing homelessness which provides a prepackaged bag of nonperishable lunch items, a fresh sandwich and more Engages local multi‐sector partners throughout the community
Mobile Food Fair events hosted by other Healthy Pantries	2020–21	Collaboration with four other local food pantries participating in the Client Choice Program Provides mentoring support and shares resources (e.g., participant consumables and equipment) and educational materials promoting fresh produce consumption

#### 2.2.2 Organizational champion

HHB's Executive Director, Nikki Rincon, has been the organizational champion for HHB, spearheading the partnership with Be Well Baytown. In this role, Ms. Rincon serves on the Steering Committee and leads the food security working group. Ms. Rincon leads efforts to work with Be Well Baytown to establish relationships with local partners from other sectors such as the school district, community college, and primary care clinics.

#### 2.2.3 Institutional investments to build HHB capacity

Since 2017, Be Well Baytown and HHB have maintained a formal partnership through a service agreement. HHB implements programs to achieve specific goals developed in collaboration with the Steering Committee. At the start of the partnership, HHB's work centered around the evidence‐based intervention: healthy food initiatives in food pantries^29^; specific goals included: (1) increasing opportunities to deliver food access programs, (2) serving larger numbers of families with food assistance including fresh produce, (3) expanding services to include nutrition education, and (4) building collaborations with local social/health agencies and as other local food assistance CBOs to improve healthy food access.

To support the expansion of HHB capacity to meet their goals, Be Well Baytown provided direct funding for program implementation, which facilitated expansion of the HHB staff and resolution of financial considerations that come with expanded capacity such as implementation of mobile food fair events and the organizational and physical infrastructure needed for increased food storage and distribution. The Be Well Baytown team maintained consistent, bi‐weekly meetings with HHB to work through financial, logistical, and other barriers to achieving stated goals.

Be Well Baytown offered technical expertise in program evaluation and data management to ensure consistent accounting of HHB activities. Other technical assistance centered around promotions and communications, including both the development of marketing materials for events and services, as well as public relations support, leading to multiple newspaper articles about the organization. To support ongoing sustainability of HHB's expanded capacity, Be Well Baytown offered organizational support such as streamlining policies and procedures, and grant writing training to enable future capital.

#### 2.2.4 Local intersectoral partner organizations

One on one meetings with HHB as well as Steering Committee meetings and workgroups created opportunities for HHB to build relationships with other local partners across diverse sectors. Partnerships with other organizations and development of an extensive volunteer network supported expansion of food distribution and healthy food access efforts throughout the community. Local partners played different roles depending on their strengths and resources. For example, partners such as the parks and recreation department had the space and infrastructure to host mobile food fairs, allowing HHB to further its geographical reach. Local partners also supported the development of tailored food security programs, such as one for students at a local community college campus, and another for patients experiencing FI at a local primary care clinic. HHB has served as a key organization for the development of Baytown's Food Security Network, a collaborative coalition of local organizations to identify, prioritize, and address community food access needs. One of their first accomplishments was expanding the volunteer network, which has supported food access programs across the community.

#### 2.2.5 Capacity evaluation metrics

Four measures of capacity were assessed for the programs implemented by HHB: (1) Reach defined as the average number of families served per quarter in each fiscal year, (2) Outreach defined as number of mobile food events organized each year, (3) Food Distributed defined as pounds of food distributed each year, and (4) Number and type of HHB community organization partners. HHB reported total families served, pounds of food distributed, and number of outreach/mobiles events per month to the Be Well Baytown team. The data was then collated and submitted with quarterly reports on progress, partnerships, and program implementation. Capacity measures were examined with descriptive statistics. Open text narratives provided in the quarterly reports were qualitatively reviewed, and meetings with Be Well Baytown and HHB staff and leadership were conducted to gain further insights about program capacity and partnerships. This study was approved by The University of Texas MD Anderson Quality Improvement Assessment Board (QIAB). Informed consent was obtained when appropriate.

## 3 RESULTS

**FIGURE 2:**
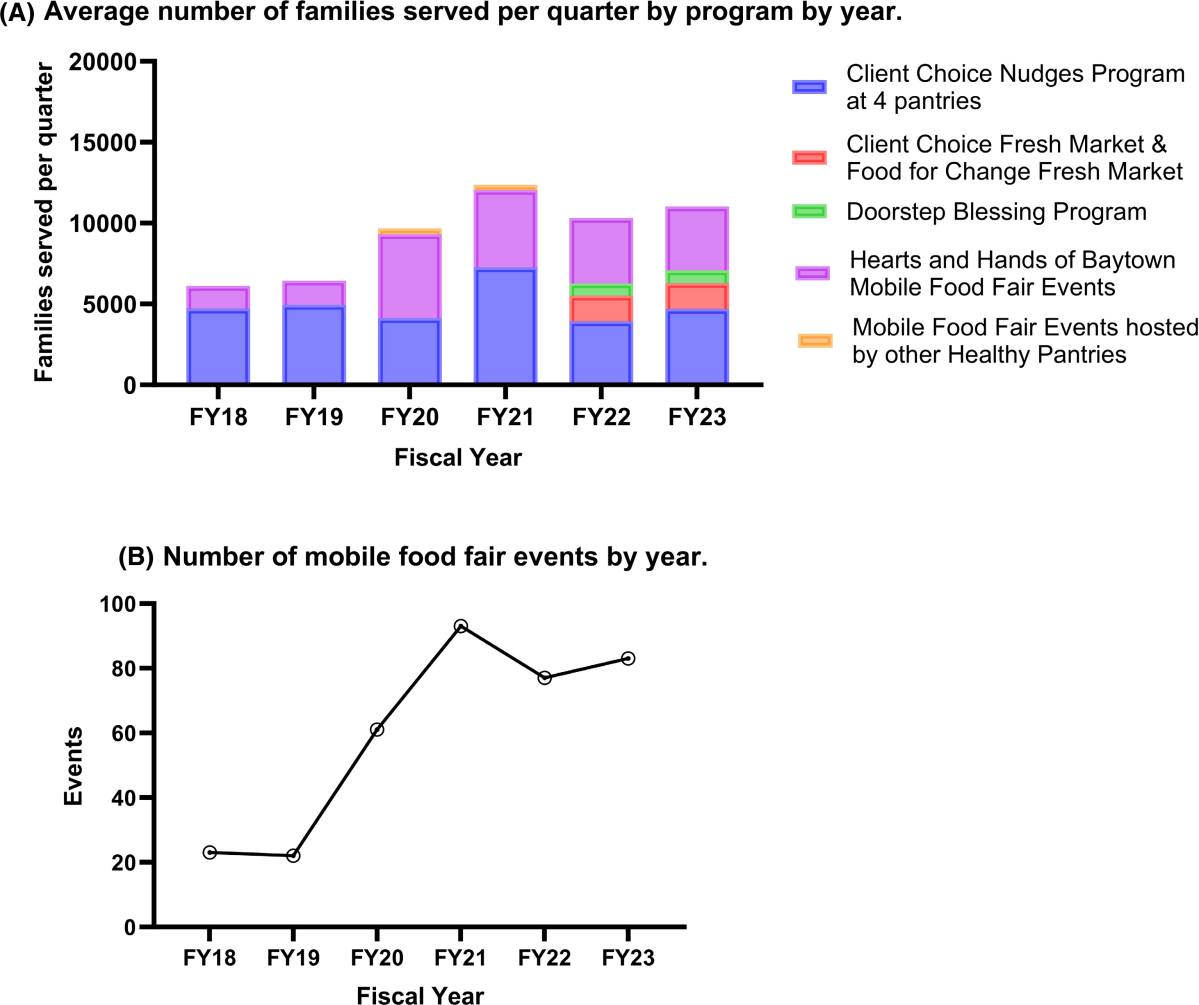
Capacity evaluation metrics.

**FIGURE 3:**
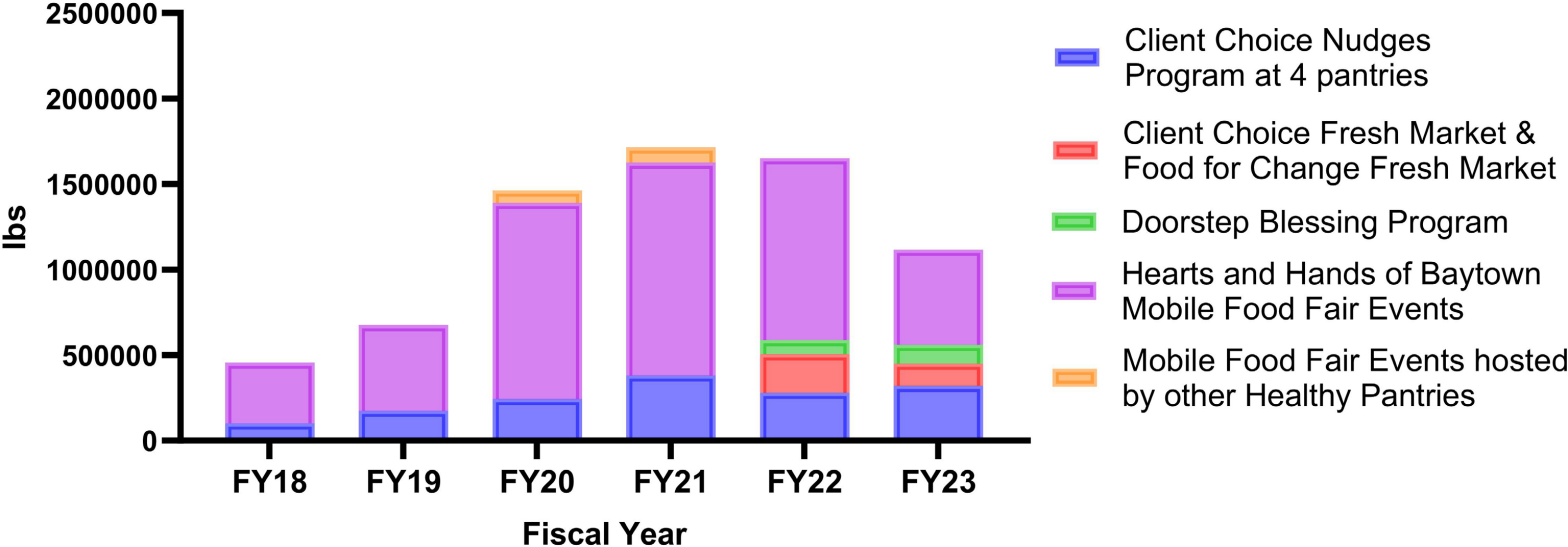
Amount of food distributed by program by year.

Prior to collaboration with Be Well Baytown, HHB hosted 10 mobile food fairs annually and distributed 126,000 pounds of food in 2017. HHB began attending Be Well Baytown Steering Committee meetings in 2018 and became the coordinating hub to organize food delivery events. From fiscal years (FY = September 1 to August 31) 2018 to 2023, HHB increased reach in average number of families served from 1397 to 3940 (Figure 2A). The program expanded to host 23 mobile food fairs in 2018, 77 in 2022 and 83 in 2023 (Figure 2B). Total pounds of food distributed rose from 354,180 to 556,003 lbs (Figure 3), with apexes of distribution and utilization occurring during the primary COVID‐19 response in 2020 and 2021. While mobile food distribution accounted for the bulk of pounds of food distributed annually across all HHB programs, total pounds of food distributed across all HHB programs from 2018 to 2023 totaled 7,073,261 lbs.

HHB reported 50 organizational partners including 27 food distribution partners (those that host mobile food events) and 9 resource referral partners (partners with processes in place to (1) refer their clients to HHB or (2) offer referrals for other services to HHB clients). Partners included the local government, area churches and senior centers, clinics, businesses, and other social service organizations. HHB staff characterized 39 of their organizational partners as “Full Collaboration” partners, meaning the organizations work together as a formal team (e.g., mutually plan, share staff/resources, and/or work under a formal agreement).

Meetings and quarterly report narratives offer further context to the trajectory and partnership data. Initial expansion was supported by additional permitting from the City of Baytown and licensing through the Houston Food Bank to expand food distribution programs. In 2019, HHB was recognized as Houston Food Bank Pantry of the Year and in 2020, HHB was chosen by the Houston Food Bank as their coordinating pantry during the pandemic to ensure food is accessible to communities during the COVID‐19 pandemic. During the pandemic response, HHB expanded its Doorstep Blessings program to reach additional families and individuals affected by the pandemic, collaborating with a local food delivery service to enhance reach during social distancing.

## 4 DISCUSSION

MD Anderson's Be Well Communities initiative aims to enhance the capacity of local food assistance CBOs in the context of cancer prevention. The case study results demonstrate that HHB was successful in achieving intended short‐term goals of increasing access to healthy food through increased reach and food distribution. This work is relevant for other CCC's and hospital systems that aim to re‐align priorities to address FI among their patients and within their areas of influence. Most existing efforts in cancer centers are concentrated in clinical settings to support cancer patients and survivors experiencing FI and include hospital‐based food pantry services^30^ or food pharmacies,^31^ assisting patients in applying for federal benefits with the help of a patient navigator^32^ or case manager, and providing medically tailored home‐delivered or pick‐up meals.^33^ Gany et al.^30^ examined the Memorial Sloan Kettering CCC's Immigrant Health and Cancer Disparities Service Food to Overcome Outcomes Disparities (FOOD) pantries, located in hospital‐based cancer clinics and found that FI was reduced among patients in all study groups. While promising, the authors noted the limitations of clinic‐based pantries with regard to food varieties and operating hours. Partnering with existing food assistance CBOs may help offset these limitations and address the needs of populations that have limited interactions with the healthcare system. The Be Well Communities approach emphasizes local partnership building to enhance the reach of food access programming into different domains of community life. HHB created resource referral partnerships with multiple Baytown‐area organizations, including developing a Food Rx Program with the local Harris County safety net healthcare system. This approach may be utilized for building healthcare‐community ecosystems around food security to connect patients across diagnoses with food access programs.

There are also examples of healthcare system‐led efforts delivered in conjunction with community partners to improve food access in the general (non‐patient) community.^34^, ^35^, ^36^, ^37^ Austin et al.^38^ describe the COE efforts at the Herbert Irving Comprehensive Cancer Center during COVID‐19 through a qualitative assessment of community organization partners during the primary pandemic response. Although no food assistance CBOs were engaged in key informant interviews, the authors observed that many community partners (such as churches) shifted to FI mitigation programs, partnering with food suppliers in the area to open food pantries. This work highlights the importance of partnership with existing community assets to address FI and emphasizes the need to integrate food assistance programs as activated community partners in the context of CCC COE programming.

The collaborative approach offered by the Be Well Communities model aims to create an enabling environment for long‐term systemic growth, which is responsive to abrupt changes in food access. In the current case study, investments in HHB prior to the pandemic allowed for rapid growth to address pandemic‐related spikes in FI and created a ready‐partner for other organizations in the area to expand food distribution efforts. This is evident in the trajectory data, which shows spikes in food distribution events as well as total pounds of food distributed during the height of pandemic response, and an expected decrease from 2022 to 2023 as the pandemic eased.

The Be Well Communities approach prioritizes sustainability through capacity building and interconnectedness with other local organizations as partners. HHB's intersectoral collaborations and additional fundraising efforts have positioned the organization for long‐term sustainability. Two additional sustainability mechanisms include a communication campaign to increase awareness of local food resources, and a community assistance navigator program to link clients to public benefits.

One strength of this case study is the relatively long data collection period, 6 years, which allowed for tracking the trajectory of the HHB over time, including before and after a pandemic that influenced food access. However, data from a single CBO is reported, limiting generalizability. Data on FI vis‐à‐vis diet quality/types of food were not collected, which is another limitation. Prospective studies at the individual level may elucidate the long‐term impact of food access efforts on household FI, diet and diet‐attributable disease risk. Future evaluations on nutritional quality and types of food supplied by CBOs can help determine whether the food distribution initiatives effectively align with dietary recommendations for cancer prevention.

## 5 CONCLUSION

CCC COE efforts that involve local capacity building and community partnerships are necessary to address FI across the cancer continuum. Collaborations have the potential to enhance the efforts of CBOs, facilitate shared knowledge transfer, elevate local decision‐making, and expand equitable services. Community coalition‐driven program implementation and collaborations with food assistance CBOs offers a mission‐bridging approach that other CCCs may leverage to engage with their communities on FI beyond the acute needs of patients.

## AUTHOR CONTRIBUTIONS

**Preena Loomba:** Conceptualization (equal); formal analysis (equal); methodology (equal); visualization (equal); writing – original draft (lead); writing – review and editing (equal). **Margaret R. Raber:** Conceptualization (equal); formal analysis (equal); methodology (equal); project administration (lead); visualization (equal); writing – review and editing (equal). **Mayra Aquino:** Data curation (supporting); project administration (supporting); validation (supporting). **Nikki Rincon:** Project administration (lead); validation (supporting). **Lori Rumfield:** Project administration (supporting). **Karen M. Basen‐Engquist:** Conceptualization (supporting); funding acquisition (supporting); project administration (supporting); writing – review and editing (supporting). **Ruth Rechis:** Conceptualization (supporting); funding acquisition (lead); project administration (lead); validation (lead); writing – review and editing (supporting).

## FUNDING INFORMATION

The project was supported by The University of Texas MD Anderson Cancer Center's Moon Shots Program, which supports the Cancer Prevention & Control Platform.

## CONFLICT OF INTEREST STATEMENT

The authors have no conflicts of interest to declare.

## PRÉCIS

The purpose of this case study is to summarize learning from supporting food assistance CBOs to effectively implement evidence‐based food insecurity mitigation programs and to better understand how to optimize use of community‐driven food security programs aligned to Comprehensive Cancer Center's (CCC) community outreach and engagement (COE) efforts. The case study demonstrates a successful community partnership strategy integrating food security programs into comprehensive cancer prevention efforts that improved the capacity of a local food pantry, resulted in expanded reach, increased food distribution, and more collaborations with partners from 2018 to 2023 offering a model for other CCCs.

## Data Availability

Data availability upon request to the corresponding author.

## References

[1] Popay J . Unequal lives: health and socioeconomic inequalities—by Graham, H., community health and wellbeing: action research on health inequalities‐ by Cropper, S., Porter, A., Williams, G., Carlisle, S., Moore, R., O.Neill, M., Roberts, C. and Snooks, H. and challenging health inequalities. Sociol Health Illn. 2008;30:478‐481.

[2] Graham H . Social determinants and their unequal distribution: clarifying policy understandings. Milbank Q. 2004;82(1):101‐124. 15016245 10.1111/j.0887-378X.2004.00303.x PMC2690205

[3] Sanchez JI , Adjei BA , Randhawa G , et al. National Cancer Institute‐funded social risk research in cancer care delivery: opportunities for future research. J Natl Cancer Inst. 2022;114(12):1628‐1635. 36073952 10.1093/jnci/djac171 PMC9949593

[4] Gany F , Lee T , Ramirez J , et al. Do our patients have enough to eat?: food insecurity among urban low‐income cancer patients. J Health Care Poor Underserved. 2014;25(3):1153‐1168. 25130231 10.1353/hpu.2014.0145 PMC4849892

[5] Gany F , Leng J , Ramirez J , et al. Health‐related quality of life of food‐insecure ethnic minority patients with cancer. J Oncol Pract. 2015;11(5):396‐402. 26286100 10.1200/JOP.2015.003962 PMC4575404

[6] Berger MH , Lin HW , Bhattacharyya N . A national evaluation of food insecurity in a head and neck cancer population. Laryngoscope. 2021;131(5):E1539‐E1542. 33098320 10.1002/lary.29188

[7] Zheng Z , Jemal A , Tucker‐Seeley R , et al. Worry about daily financial needs and food insecurity among cancer survivors in the United States. J Natl Compr Cancer Netw. 2020;18(3):315‐327. 10.6004/jnccn.2019.7359 32135509

[8] Matthew RP , Hales LJ , Burke MP , Coleman‐Jensen A . Household Food Security in the United States in 2022. U.S. Department of Agriculture, Economic Research Service; 2023:ERR‐325.

[9] Olson CM . Nutrition and health outcomes associated with food insecurity and hunger. J Nutr. 1999;129(2):521S‐524S. 10064322 10.1093/jn/129.2.521S

[10] Pérez‐Escamilla R . Food security and the 2015‐2030 sustainable development goals: from human to planetary health: perspectives and opinions. Curr Dev Nutr. 2017;1(7):e000513. 29955711 10.3945/cdn.117.000513 PMC5998358

[11] Wang L , Du M , Cudhea F , et al. Disparities in health and economic burdens of cancer attributable to suboptimal diet in the United States, 2015–2018. Am J Public Health. 2021;111(11):2008‐2018. 34648383 10.2105/AJPH.2021.306475 PMC8630501

[12] Raber M , Jackson A , Basen‐Engquist K , et al. Food insecurity among people with cancer: nutritional needs as an essential component of care. J Natl Cancer Inst. 2022;114(12):1577‐1583. 36130287 10.1093/jnci/djac135 PMC9745434

[13] Rock CL , Thomson C , Gansler T , et al. American Cancer Society guideline for diet and physical activity for cancer prevention. CA Cancer J Clin. 2020;70(4):245‐271. 32515498 10.3322/caac.21591

[14] Rock CL , Thomson CA , Sullivan KR , et al. American Cancer Society nutrition and physical activity guideline for cancer survivors. CA Cancer J Clin. 2022;72(3):230‐262. 35294043 10.3322/caac.21719

[15] Larson N , Laska MN , Neumark‐Sztainer D . Food insecurity, diet quality, home food availability, and health risk behaviors among emerging adults: findings from the EAT 2010–2018 study. Am J Public Health. 2020;110(9):1422‐1428. 32673120 10.2105/AJPH.2020.305783 PMC7427214

[16] Oliver TL , McKeever A , Shenkman R , Diewald L . Barriers to healthy eating in a community that relies on an emergency food pantry. J Nutr Educ Behav. 2020;52(3):299‐306. 31708426 10.1016/j.jneb.2019.10.005

[17] Greenhalgh T , Jackson C , Shaw S , Janamian T . Achieving research impact through Co‐creation in community‐based health services: literature review and case study. Milbank Q. 2016;94(2):392‐429. 27265562 10.1111/1468-0009.12197 PMC4911728

[18] Noel L , Phillips F , Tossas‐Milligan K , et al. Community‐academic partnerships: approaches to engagement. Am Soc Clin Oncol Educ Book. 2019;39:88‐95. 31099695 10.1200/EDBK_246229 PMC6543849

[19] Rechis R , Oestman KB , Caballero E , et al. Be well communities™: mobilizing communities to promote wellness and stop cancer before it starts. Cancer Causes Control. 2021;32(8):859‐870. 34037915 10.1007/s10552-021-01439-9 PMC8236479

[20] Rechis R , Oestman KB , Walsh MT , Love B , Hawk E . Be well™ acres homes: a community‐driven, evidence‐based approach to reduce health inequities through sustained cross‐sector partnership. Cancer Causes Control. 2023;35(4):611‐622. 37979072 10.1007/s10552-023-01818-4 PMC10960758

[21] Oestman K , Rechis R , Williams PA , et al. Reducing risk for chronic disease: evaluation of a collective community approach to sustainable evidence‐based health programming. BMC Public Health. 2024;24(1):240. 38245669 10.1186/s12889-024-17670-3 PMC10799505

[22] "ACS Demographic and Housing Estimates ." 2022. https://data.census.gov/table/ACSDP1Y2022.DP05?q=Baytown&g=160XX00US4806128&y=2022.

[23] "Selected Characteristics of the Native and Foreign‐Born Populations ." 2022. https://data.census.gov/table/ACSST5Y2022.S0501?q=Baytown&g=160XX00US4806128&y=2022.

[24] "Selected Characteristics of Health Insurance Coverage in the United States ." 2022. https://data.census.gov/table/ACSST1Y2022.S2701?g=160XX00US4806128.

[25] "Selected Characteristics of the Total and Native Populations in the United States .". 2022. https://data.census.gov/table/ACSST1Y2022.S0601?q=Baytown&g=160XX00US4806128&y=2022.

[26] "Food Stamps/Supplemental Nutrition Assistance Program (SNAP) ." 2022. https://data.census.gov/table/ACSST1Y2022.S2201?q=SNAP/Food.

[27] Conduent Healthy Communities Institute . Food Insecurity Index. 2023. Accessed June 10, 2024. https://help.healthycities.org/hc/en‐us/articles/360060385334‐What‐is‐the‐Food‐Insecurity‐Index.

[28] Goodman RM , Speers MA , McLeroy K , et al. Identifying and defining the dimensions of community capacity to provide a basis for measurement. Health Educ Behav. 1998;25(3):258‐278. 9615238 10.1177/109019819802500303

[29] University of Wisconsin Population Health Institute . Healthy food initiatives in food pantries. County Health Rankings & Roadmaps (CHR&R) Web site. Accessed November 09, 2023. https://www.countyhealthrankings.org/strategies‐and‐solutions/what‐works‐for‐health/strategies/healthy‐food‐initiatives‐in‐food‐pantries.

[30] Gany F , Melnic I , Wu M , et al. Food to overcome outcomes disparities: a randomized controlled trial of food insecurity interventions to improve cancer outcomes. J Clin Oncol. 2022;40(31):3603‐3612. 35709430 10.1200/JCO.21.02400 PMC9622577

[31] Lindau Laboratory at the University of Chicago . Feed1st Food Pantry Toolkit: How to Launch an Open Access Food Pantry in Your Organization. University of Chicago; 2019.

[32] Gany F , Ramirez J , Nierodzick ML , McNish T , Lobach I , Leng J . Cancer portal project: a multidisciplinary approach to cancer care among Hispanic patients. J Oncol Pract. 2011;7(1):31‐38. 21532808 10.1200/JOP.2010.000036 PMC3014508

[33] Gany FM , Pan S , Ramirez J , Paolantonio L . Development of a medically tailored hospital‐based food pantry system. J Health Care Poor Underserved. 2020;31(2):595‐602. 33410795 10.1353/hpu.2020.0047 PMC8073793

[34] Lundeen EA , Siegel KR , Calhoun H , et al. Clinical‐community partnerships to identify patients with food insecurity and address food needs. Prev Chronic Dis. 2017;14:E113. 29144894 10.5888/pcd14.170343 PMC5695644

[35] Hiatt RA , Sibley A , Fejerman L , et al. The San Francisco cancer initiative: a community effort to reduce the population burden of cancer. Health Aff (Millwood). 2018;37(1):54‐61. 29309234 10.1377/hlthaff.2017.1260

[36] Memorial Sloan Kettering Cancer Center . Food to Overcome Outcome Disparities (FOOD). Accessed Oct 26, 2023. https://www.mskcc.org/departments/psychiatry‐behavioral‐sciences/immigrant‐health/addressing‐socioeconomic‐determinants‐health/food‐overcome‐outcome‐disparities.

[37] Public Health Institute . Making Food Systems Part of Your Community Health Needs Assessment. 2016. Public Health Institute.

[38] Austin JD , Burke K , Argov EJL , et al. Experience of a National Cancer Institute‐designated community outreach and engagement program in supporting communities during the COVID‐19 pandemic. J Community Health. 2022;47(5):862‐870. 35819548 10.1007/s10900-022-01115-2 PMC9751522

